# Visualization of a Mammalian Mitochondrion by Coherent X-ray Diffractive Imaging

**DOI:** 10.1038/s41598-017-01833-x

**Published:** 2017-05-12

**Authors:** Yoonhee Kim, Chan Kim, Ou Young Kwon, Daewoong Nam, Sang Soo Kim, Jae Hyun Park, Sunam Kim, Marcus Gallagher-Jones, Yoshiki Kohmura, Tetsuya Ishikawa, Changyong Song, Giyoong Tae, Do Young Noh

**Affiliations:** 1 Department of Physics and Photon Science & School of Materials Science and Engineering, Gwangju Institute of Science and Technology, Gwangju, 61005 Korea; 2 European XFEL, Schenefeld, 22869 Germany; 3 Department of Physics, Pohang University of Science and Technology, Pohang, 37673 Korea; 4 Pohang Accelerator Laboratory, Pohang University of Science and Technology, Pohang, 37673 Korea; 5 RIKEN SPring-8 Center, Kouto 1-1-1, Hyogo, 679-5148 Japan; 6 Department of Physics and Astronomy and California NanoSystems Institute, University of California Los Angeles, California, 90095 USA

## Abstract

We report a three dimensional (3D) quantitative visualization of a mammalian mitochondrion by coherent x-ray diffractive imaging (CXDI) using synchrotron radiation. The internal structures of a mitochondrion from a mouse embryonic fibroblast cell line (NIH3T3) were visualized by tomographic imaging at approximately 60 nm resolution without the need for sectioning or staining. The overall structure consisted of a high electron density region, composed of the outer and inner membranes and the cristae cluster, which enclosed the lower density mitochondrial matrix. The average mass density of the mitochondrion was about 1.36 g/cm^3^. Sectioned images of the cristae reveal that they have neither a baffle nor septa shape but were instead irregular. In addition, a high resolution, about 14 nm, 2D projection image was captured of a similar mitochondrion with the aid of strongly scattering Au reference objects. Obtaining 3D images at this improved resolution will allow CXDI to be an effective and nondestructive method for investigating the innate structure of mitochondria and other important life supporting organelles.

## Introduction

Over the last few decades, there has been enormous effort to visualize the internal structures of cellular organelles utilizing a variety of microscopy techniques such as optical, fluorescence, and electron microscopies^1–10^. Most methods, however, have limitations either in resolution or are disruptive to the sample under investigation. The resolution of conventional optical microscopies is fundamentally limited to several hundred nanometers due to long wavelengths used, following Abbe’s principle. This is not sufficient to observe most sub-cellular structures. Fluorescence microscopies can overcome the diffraction limit through the use of extensive labeling and computational techniques, however labeling can interfere with quantitative visualization and observed structures lack an overall cellular context. Much higher spatial resolution is available in electron microscopies but thick specimens have to be sectioned which disrupts their internal structure, and in addition most of them need to be stained with heavy metals, which complicates image interpretation^11–13^. Moreover, all these methods suffer from the aberration issues caused by various types of lenses involved in microscopes.

Coherent x-ray diffractive imaging (CXDI), which has the ability to visualize non crystalline specimens nondestructively at about 10~20 nm resolution, offers an alternative to conventional microscopy methods^14–19^. In CXDI, two-dimensional diffraction patterns are directly recorded from x-rays that penetrate through a specimen and diffract. These patterns are phased directly in iterative phase retrieval algorithms to produce corresponding projection images. CXDI is a lens-free technique, since images are reconstructed computationally from the measured diffraction signal without employing any image forming lenses. Two-dimensional (2D) projection images at various angles can also be assembled to reconstruct a three-dimensional (3D) image using the tomography principle^20, 21^. 3D imaging of human chromosome^22^, mammalian nucleus^23^ and several unicellular organelles^17, 24, 25^ have been successfully demonstrated using CXDI.

In this work, we demonstrate the feasibility of CXDI for imaging the internal structure of a mammalian mitochondrion three dimensionally. Mitochondrion is an essential eukaryotic organelle involved in diverse cellular processes including; energy generation, senescence, cell growth, reproduction, genetics and maternal inheritance^26–29^. Given their central role in such a diverse array of cellular function the structure of mitochondria has been the subject of intensive study^26–29^. The functions of mitochondria are known to be closely related to their intricate internal structure, although this depends on the species, organs, and cells in which they reside^12, 30^. Cristae, the distinct invaginations of inner membrane of mitochondria, for example, the site of the electron transport chain and ATP synthase that generate ATP, which is the dominant source of intracellular energy^26–29^. The complicated internal structure of the cristae enlarges surface area and increases the enzymatic reaction sites for ATP synthesis.

The internal ultra-structure of mitochondria is typically classified into three shapes: normal (tubule and branch), non-tubular, and swollen shape^31^. The structures have only been observed, however, from samples that have been sectioned and stained, which hinders precise measurement and introduces artifacts making interpretation of their density maps difficult. In this work, we illustrate the feasibility of visualizing unstained and unsectioned mitochondria by applying CXDI to a mitochondrion of NIH3T3 (primary mouse embryonic fibroblasts cell line) which is a standard mammalian fibroblast cell line. A 3D image reconstructed by tomographically assembling 2D projection images at various angles was used to reconstruct a quantitative 3D density map. Furthermore, a high resolution 2D projection image was obtained using strongly scattering Au reference objects^32, 33^ that amplify the diffraction signal.

## Results and Discussion

### 2D projection imaging of a mammalian mitochondrion

**Figure 1:**
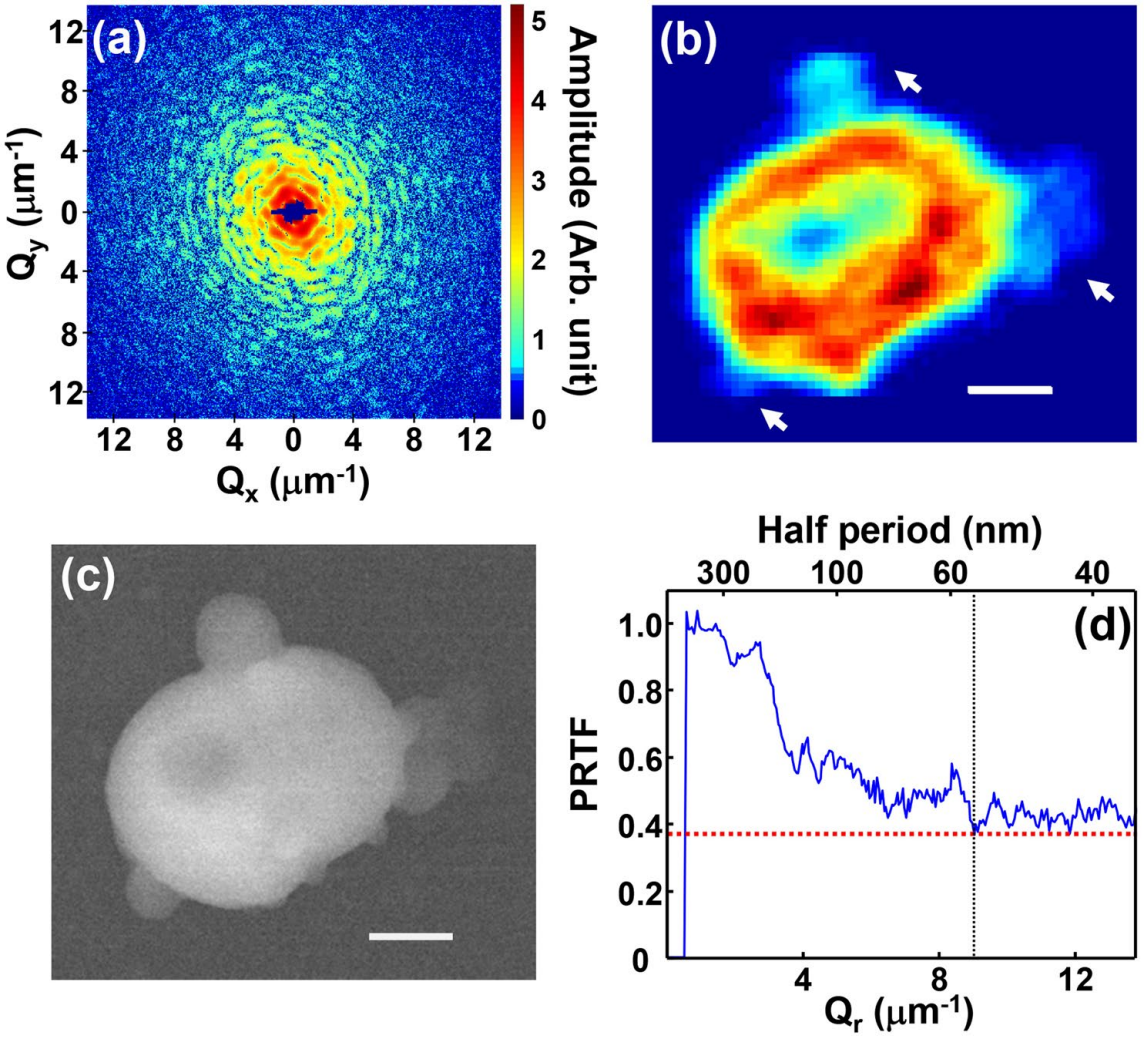
(**a**) Coherent x-ray diffraction pattern recorded from an isolated mitochondrion obtained at zero degree projection angle presented on logarithmic scale. (**b**) 2D CXDI projection image of the mitochondrion reconstructed using the data shown in (**a**). The particles indicated by the arrows are undesired dusts. (**c**) SEM image of the mitochondrion which represents mostly its surface morphology. The scale bars in (**b**,**c**) are 400 nm. (**d**) PRTF evaluated from the iterative phase retrieval process. The dotted vertical line indicates the position where PRTF saturates, which was used to estimate the spatial resolution.

Figure 1(a,b) show the diffraction amplitude and the reconstructed projection image of a mitochondrion obtained at zero degree projection angle. The coherent x-ray diffraction measurements were performed at BL29XU at SPring-8, a third generation synchrotron source in Japan. The energy of the x-ray beam was fixed at 5 keV and the specimen was confined within the coherent volume of the incident x-ray beam, ensuring that the recorded far-field diffraction pattern was the exact square of the Fourier transform of the electron density integrated along the incident x-ray beam direction (projected density). The shape of the projection image in Fig. 1(b) matches well with the morphology shown in the scanning electron microscopy (SEM) image in Fig. 1(c). However, the size of mitochondrion in the SEM image is slightly smaller than retrieved image shown in Fig. 1(b). We suspect that some shrinkage occurred during the SEM imaging process due to the high vacuum, 4.4 × 10^−4^ Pa, and electron beam intensity, with acceleration voltage of 10 kV, used during this measurement rather than the x-ray exposure. We confirmed that there was no significant change due to the x-ray irradiation by measuring the zero degree diffraction profile again after a long exposure (total of 470 min.) and reconstructing the projected image (Supplementary Fig. S1). We also observed the shrinkage of the image of a mitochondrion by an SEM measurement.

The CXDI image shown in Fig. 1(b), retrieved by the guided hybrid-input-output (GHIO) algorithm^34^, provides traces of the structure including a low-density internal region surrounded by a higher density exterior, however it is difficult to make such interpretations from the two dimensional image since it represents the electron density map integrated along the direction of the incident x-ray beam. This makes quantitative interpretation of the projection image difficult. The surrounding outer region with high electron density is composed of mitochondrial membranes and cristae. Several bulged objects indicated by arrows in Fig. 1(b) are undesired dust particles which were attached to mitochondrion during specimen extraction or deposition on to the Si_3_N_4_ substrate. The bulged objects were mostly composed on carbon and oxygen as confirmed in an scanning electron microscopy energy dispersive spectroscopy (SEM-EDS) analysis, and it is likely that they were cell remnants of remained during the sample preparation.

The image pixel size in Fig. 1(b), set by the extent of the diffraction intensity shown in Fig. 1(a), is about 36 nm. The spatial resolution was conservatively estimated to be 55 nm using the phase retrieval transfer function (PRTF)^35^ shown in Fig. 1(d). Although the value of PRTF was over the typical resolution criterion, 1/*e*, employed in most CXDI imaging, the decrement stagnated after the half period of 55 nm which is marked with a dotted line in the figure where we set the spatial resolution. Although there is another degressive edge near 150 nm, we have selected the 55 nm since the PRTF is closer to 1/e. In the reconstructed images both in 2D and 3D, we found several features smaller than 100 nm concluded that 55 nm edge was the more reasonable choice.

### 3D tomographic visualization and analysis on mammalian mitochondrion

For 3D visualization of the mitochondrion, we obtained a set of 26 diffraction profiles measured at incrementing projection angles ranging from −63.43° to 69.44° by rotating the sample around an axis perpendicular to the x-ray beam direction. These angles were selected specifically to follow an equally sloped trajectory necessary for use in the equally sloped tomography (EST) method^20, 21^. EST minimizes the number of projection images necessary for accurate 3D reconstruction, which reduces data acquisition time and subsequently reduces the radiation dose imparted to the specimen. It took about 11.5 hours to acquire the entire tomography series, which corresponds to about 4.26 × 10^8^ *Gy* of radiation dose (see methods). Judging from the studies on the radiation damage threshold^36^, this amount of radiation dose would not cause significant radiation damage to features of interest at the observed resolution.

**Figure 2:**
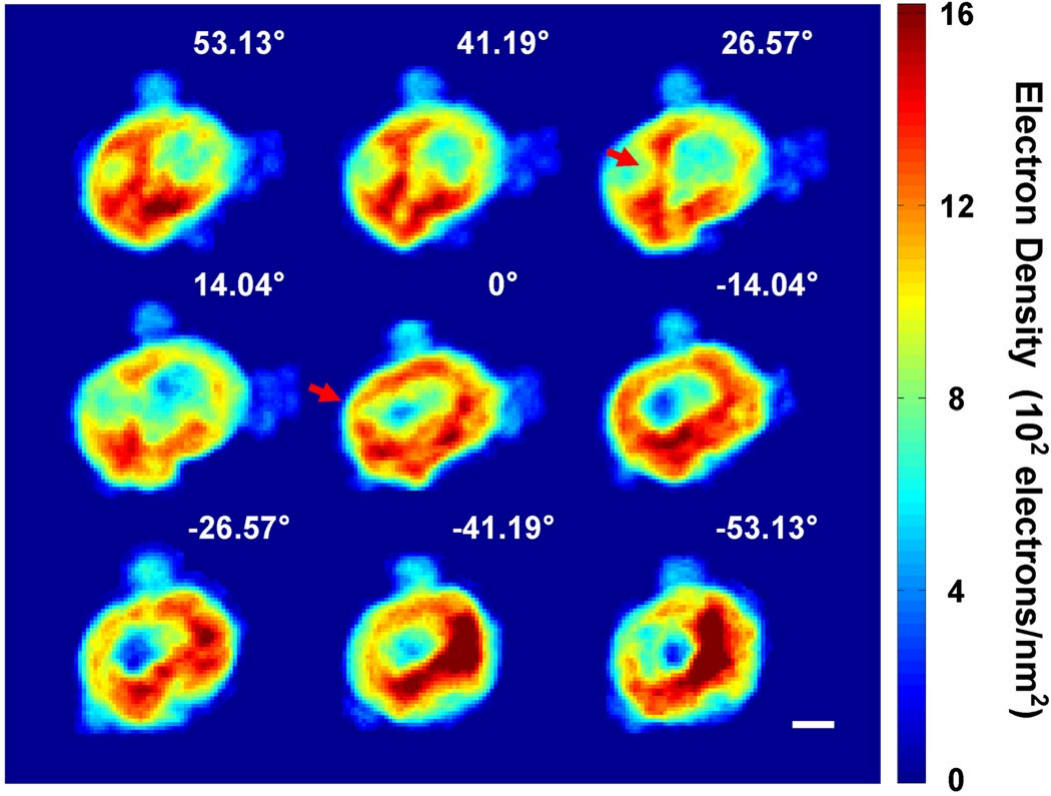
Representative projection 2D CXDI images selected from total of 26 images. They are reconstructed from the diffraction profiles measured at the corresponding projection angles, and represent the electron density map integrated along the beam direction. As the projection angle changes, the image of the inner low density area changes consistently. Some images show noticeable density variations over two or three pixels as indicated by an arrow. The scale bar is 400 nm.

Shown in Fig. 2 are 9 representative 2D projection images reconstructed from the diffraction patterns measured at the corresponding angles. All 26 diffraction data and reconstructed images are shown in the supplementary information (Supplementary Fig. S2). The images changed consistently as the projection angle was incremented. Regions exhibiting significant density variations over two or three pixels, as marked by arrows in the images, indicate the existence of small structures separating low density hollows. To reconstruct a 3D image from the projection CXDI images using the EST tomographic algorithm^20, 21^, we aligned all 26 projection images along the rotation axis and matched the center of mass of each projection. Detailed procedures for data measurement and image reconstruction are described in the methods section. Based on the PRTF analysis, we estimate the resolution of the 3D image to be about 60 nm (Supplementary Fig. S3) similar to that of the 2D image (Fig. 1(d)).

**Figure 3:**
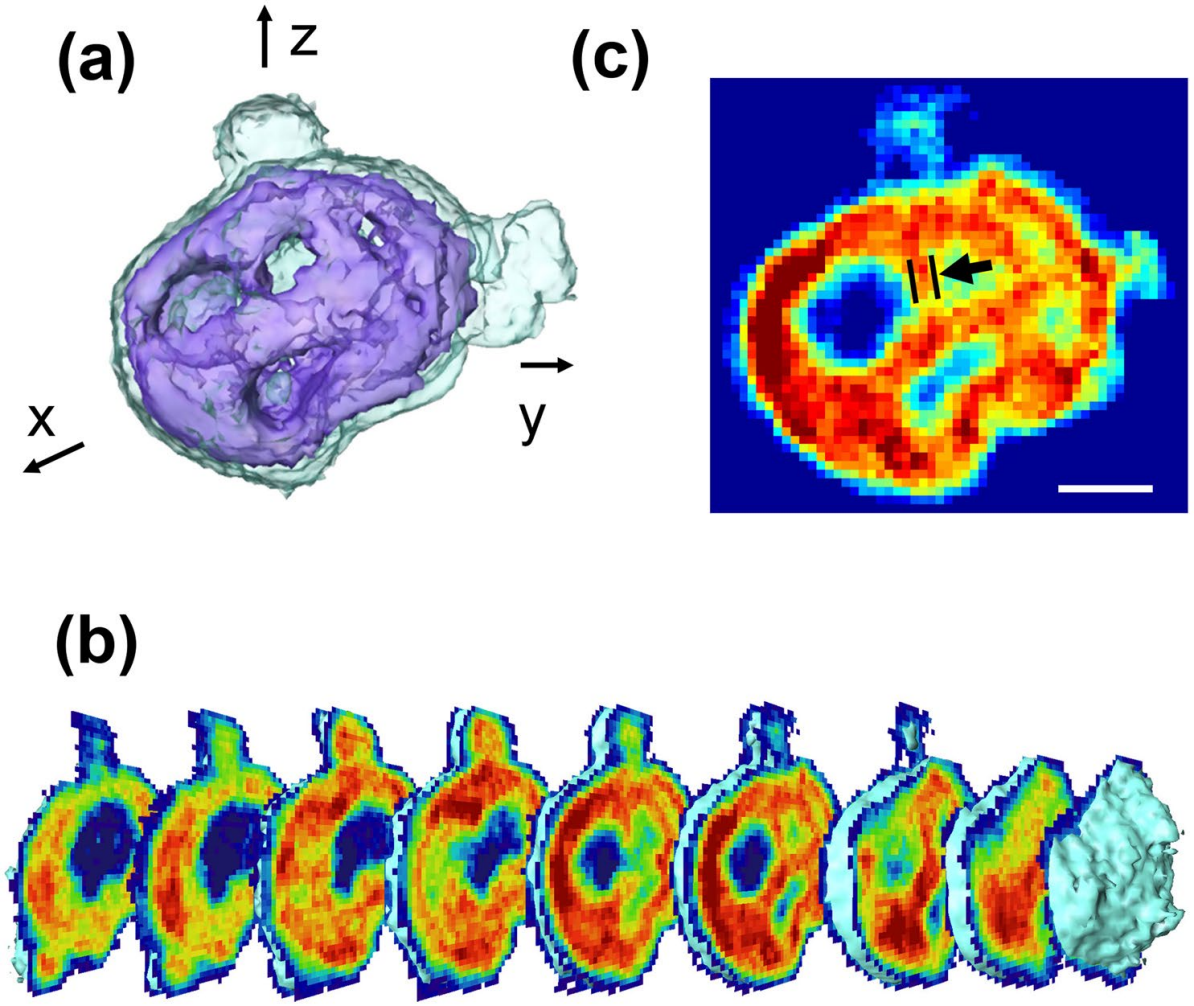
(**a**) 3D rendering of the mitochondrial morphology. (**b**) Sectioned images of the 3D mitochondrion tomogram cut in the y-z plane obtained at 109 nm intervals that illustrate the progressive change of the internal structure. (**c**) One of the sectioned images shown in (**b**) exhibiting internal structures well. The arrow indicates the width of a structure stemming from the exterior region, which is about 100 nm. The scale bar is 400 nm.

A 3D rendering of the reconstructed tomographic image illustrated in Fig. 3(a) shows that the mitochondrion was ellipsoidal in shape and about 1.7 (1.2) *μ*m in length in the major (minor) axis direction. The volume of retrieved mitochondrion, excluding the bulged objects, was 2.83 *μ*m^3^. The total number of electrons in the mitochondrion was estimated to be 1.24 × 10^12^ and the mass density deduced from this was 1.36 g/cm^3^. When we isolate mitochondria from cells, the mass density of each mitochondrion was estimated to be of order 1.15 g/cm^3^ ^37^, about 84% of the estimation from the tomography analysis. The estimation of the electron density, the volume, the mass density, and the mitochondria isolation procedure are described in the methods section.

The rendering shows that the high electron density outer region encloses a rather low density internal area and defines the mitochondrial shape. We suspect that the high density region consists of the mitochondrial membranes (inner and outer) and cristae stemming from them, although each face of cristae, whose typical thickness is reported to be about 12–40 nm^12^, is too small to be resolved at the current resolution. The low density inner region supposedly includes semifluid matrix containing mitochondrial DNA molecules, ribosomes as well as various kinds of enzymes involved in mitochondrial function. When we define a density cutoff for the mitochondrial matrix at 53.4% of the maximum density, *ρ*_*max*_ of 1443.6 electrons/nm^3^, the volume occupied by the matrix is 2.15 *μ*m^3^, about 76.0% of the whole mitochondrial volume. Although the specimen was chemically fixed to prevent morphology distortion, some of solid components might have migrated to the surface of membrane during the drying process.

Cross-sections of the 3D image sliced at 109 nm intervals are illustrated in Fig. 3(b). Each image shows the electron density map on the surface of the corresponding slice averaged over two pixels perpendicular to the surface. These sectioned images show that the cristae are neither in typical baffle shape with wide openings into the matrix nor septa shape, but rather irregular in shape. They stem from the membranes in the outside and connected internally as indicated in one of the slices shown in Fig. 3(c). The thickness of each structure which is considered as an infolding extends to about three pixels, ~100 nm as indicated by an arrow. Although we found several features smaller than 100 nm in the reconstructed images (Supplementary Fig. S4), the current resolution, about 60 nm, was not quite enough to identify the detailed layer structure of the cristae infoldings.

More detailed section images are illustrated in Supplementary Figs S5–S7 which show surface images of the sections cut in every other pixel normal to the image plane in all three directions. The cristae morphology appears differently depending on the direction and position of sections. The infolding structures are more noticeable in the slices sectioned in the y-z plane (Supplementary Fig. S5), but it is difficult to identify them in the slices cut in the x-z, and y-z planes (Supplementary Figs S6 and S7). Some cristae might be grouped together within the mitochondrial membrane to form thick crust regions. It is known that the internal structure of a mitochondrion varies a lot depending on the species, organ or tissue in which they reside^12, 30^. Moreover, it is possible that several classes of internal structures are present inside a single mitochondrion. The capability of sectioning in an arbitrary direction computationally is a great advantage of the 3D CXDI tomography as compared to TEM which requires physical thin sectioning that damages a specimen, which makes it impossible to view in other sectioning direction.

### Resolution enhancement using reference Au particles

**Figure 4:**
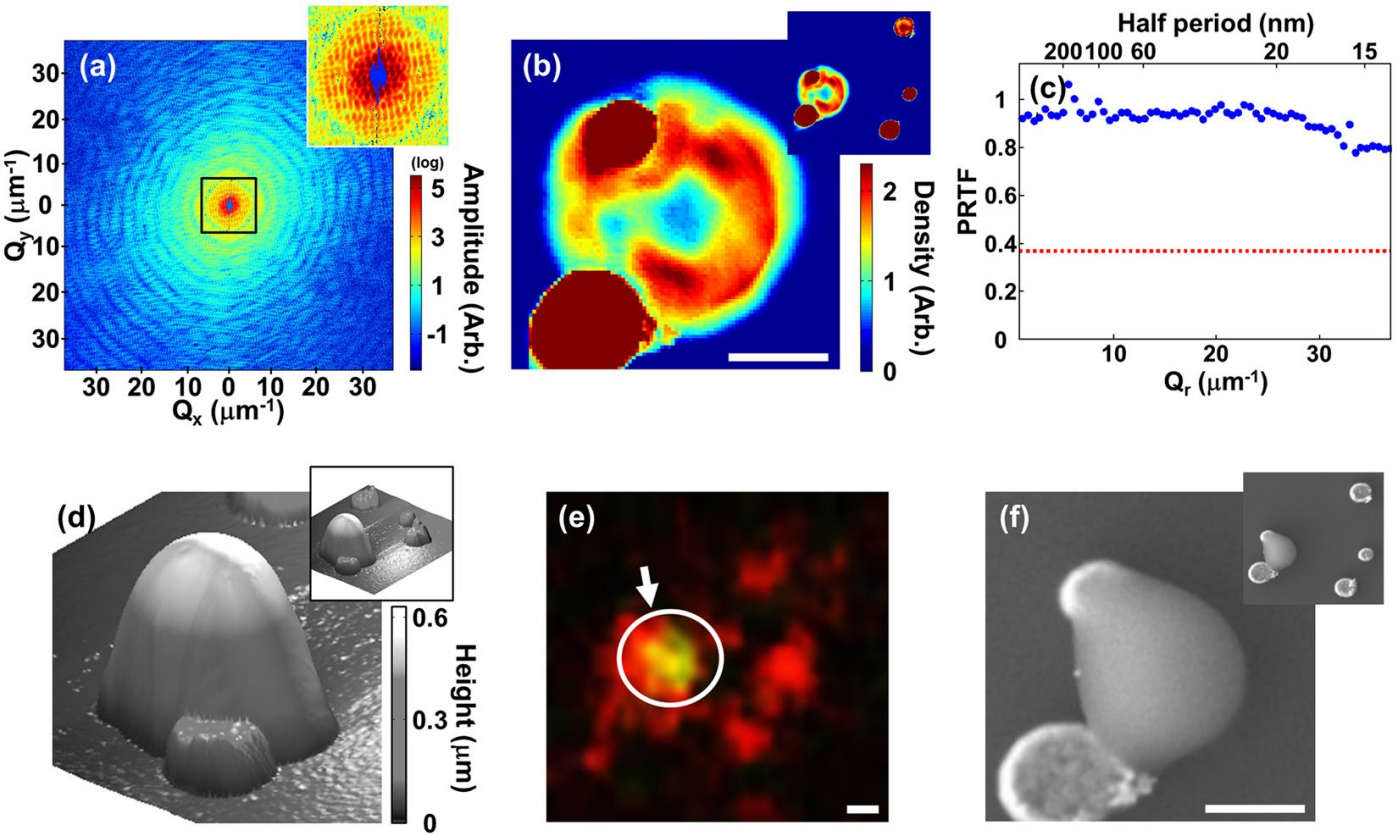
(**a**) Diffraction amplitude from a specimen with a mitochondrion together with Au reference objects. The central part is enlarged in an inset to display details of the speckle pattern. (**b**) CXDI projection image of the mitochondrion reconstructed from (**a**). The red circular objects are the Au reference objects. An image with an enlarged field of view is included in the upper right corner. (**c**) PRTF evaluated from the iterative reconstruction which indicates that the image resolution is around 14 nm. Red dotted line indicates 1/e, the resolution criterion. (**d–f**) AFM (**d**), fluorescence (**e**), and SEM (**f**) image of the same specimen. The green fluorescence emission indicated in (**e**) shows that the measured object is indeed a mitochondrion. The scale bars are 400 nm.

Although the 3D reconstruction presented above visualizes the overall internal structure of the mitochondrion, the resolution places limits on detailed, quantitative interpretation of internal features such as the cristae. The limitation in the image resolution was mainly caused by the lack of meaningful diffraction signal at high diffraction angles as in most reported CXDI experiments of biological specimens. To increase the range of diffraction signals, higher incident x-ray flux or longer exposure is necessary which inevitably leads to significant radiation damage. Recent ‘diffraction-before-destruction’ methods based on XFEL sources open a way to obtain an image at a higher resolution^38, 39^ without concern over radiation damage. Using an ultrashort intense XFEL pulse which lasts only a few tens of femtoseconds, one might expect to obtain a diffraction pattern prior to the disintegration of a specimen. It is, however, impossible to apply typical tomographic methods for 3D imaging since the specimen would be destroyed after a single 2D measurement in one direction. Recent reports including the work by Kim *et al*.^32^ suggested another method of enhancing the spatial resolution without increasing x-ray dose using reference objects. They demonstrated that the strongly scattering reference objects enhance the spatial resolution in CXDI imaging significantly. The interference signal between the reference objects and a specimen increases as the square root of the reference signal. In the meanwhile, the Poisson’s noise would also increase in a similar manner, and there is no gain in the signal-to-noise ratio. The enhancement, however, appears when the instrumental noise becomes larger than the Poisson’s shot noise of the sample object. By comparing the signal-to-noise ratio with and without the reference object, they showed that the resolution can be enhanced by using reference objects. In their simulation on a biological object with similar density as a mitochondrion, they demonstrated that Au reference objects of a few 100 nm size enhance the CDI resolution significantly. By introducing Au particles as strongly scattering reference objects near a mitochondrion specimen and increasing incident flux density by focusing x-rays employing Kirkpatrick-Baez (K-B) mirrors, we achieved a high resolution 2D image of a mitochondrion. As shown in the measured diffraction amplitude in Fig. 4(a), the diffraction signal extends across the entire area of the CCD chip. Shown in Fig. 4(b) is the CXDI image of the mitochondrion specimen together with the reference Au particles reconstructed from the data in Fig. 4(a) using a GHIO algorithm. Figure 4(c) shows the PRTF calculated from the phase retrieval iterations. It remains above 0.7 across the entire range of the spatial frequency space indicating that the image resolution is comparable to the single pixel size of 14 nm. This implied that the factor limiting the resolution is not the diffracted signal but the range of the detector. Although there exists some density variations over two to three pixels in Fig. 4(b) and the line profile across the a Au particle is sharp reflecting the improved resolution (Supplementary Fig. S8), the complex internal structures of mitochondrion were not distinguishable because it is a projected 2D image where the densities are integrated along the beam direction. It is difficult to resolve structures with weak density variations in a 2D projected image.

The CXDI image was compared to atomic force microscopy (AFM), fluorescence microscopy, and SEM images shown in Fig. 4(d–f). The AFM image shows the surface profile of the mitochondrion which matches well with the CXDI image. The mitochondrion is thicker than the Au reference objects (Fig. 4(d)), but it has lower density as shown in reconstructed image (Fig. 4(b)). The components of biological materials are mostly composed of light elements and the mitochondrion has large empty space inside. The fluorescence microscopy image shown in Fig. 4(e) identifies the mitochondrion emitting green light although the shape is not clearly distinguishable due to very limited resolution. The mitochondrion was stained with Rhodamine123. (see methods). The mitochondrion was shrunken by electron beam under vacuum as shown in the SEM image (Fig. 4(f)) which was performed after all the other imaging methods.

## Conclusion

In this work, a mammalian mitochondrion from a mouse embryonic fibroblasts cell line was visualized in 3D by using CXDI nondestructively without cutting and staining. A 3D density map of the mitochondrion was obtained quantitatively at about 60 nm spatial resolution. The mitochondrion was in an ellipsoidal shape with a low density inner matrix was enclosed by a loosely connected outer crust. The internal morphology is not in line with the typically reported regular shape such as baffle or septa structures, but rather has an irregular arrangement. Structures on the order of 100 nm in size, considered to be cristae, were observed via computational sectioning of the reconstructed volume. They were loosely connected to the outer wall composed of mitochondrial membranes. The capability of sectioning in arbitrary directions was powerful in visualizing the internal structure of the mitochondrion.

The 3D spatial resolution presented here was not quite enough to resolve the detailed structure of cristae reliably. By introducing amplifying reference objects, the spatial resolution of a 2D image was enhanced up to 14 nm. We suggest that 3D CXDI visualization with an improved resolution is feasible by employing the reference objects. We expect that the detailed internal structure will be revealed by 3D CXDI tomography with a high resolution in the near future. High resolution 3D CXDI visualization would answers various questions involved in mitochondrial functions and diseases related to mitochondria.

## Methods

### Mitochondria isolation

NIH3T3 cells, the mouse embryonic fibroblasts were cultured in petri dishes (10 pi, Corning) and submerged into Dulbecco’s modified Eagle’s medium (DMEM, GIBCO) with 10% fetal bovine serum (FBS, GIBCO) and 1% antibiotics (GIBCO). They were incubated at 36 °C in a humidified incubator with 5% CO_2_ to proliferate cells for more than 24 hours. When the cells grew and covered more than 70% of a dish, they were immersed in a solution of 0.25% trypsin and 0.02% ethylenediamine tetraacetic acid (EDTA) for three minutes to detach cells from the dish. The solution was neutralized using the same amount of DMEM with 10% FBS to prevent cell damage. The solution was removed by centrifuging at 4 °C, 1,500 rpm for 3 minutes, and the cell pellets detached from the dish were washed by phosphate buffered saline (PBS) to remove remaining trypsin/EDTA.

We followed the protocol of OptiPrep™ (Axis-Shield)^40^ for solution compositions and procedures to isolate mitochondria. The cells thus prepared were first suspended in a homogenization medium and homogenized using a tight-fitting Dounce homogenizer (Wheaton Type A), then centrifuged at 4 °C at 1,000 rpm for 10 minutes. We extracted supernatant and kept it separately. The remaining cell pellets were resuspended in the homogenization medium and homogenized by loose-fitting Dounce homogenizer (Wheaton Type B) and centrifuged following the same procedure as before, and we extracted supernatant again. All supernatant was put together and centrifuged. The pellets resulted from the above process, which contain mitochondria, were resuspended by the homogenization medium and used for sucrose gradient centrifugation. These mitochondria extracts were loaded on the bottom of tube for ultracentrifuge and 1.175 g/cm^3^ and 1.079 g/cm^3^ sucrose solutions were loaded step by step for sucrose gradient centrifugation. A thin layer of the homogenization medium was added on the top for protection. The solution was centrifuged at 50,000 g (g-force), 4 °C for 4 hours in no break mode. This centrifugation separated mitochondria in a thin layer in the middle of medium corresponding to 1.145 g/cm^3^ of medium density. After extracting this layer we added the same amount of PBS and centrifuged at 4 °C at a speed of 17,000 g for 15 minutes for washing. This process was repeated for three times, and the mitochondria were fixed by 10% formalin at room temperature for 30 minutes. The formalin was removed after centrifugation at 4 °C and 17,000 g for 15 minutes. The remaining mitochondria pellet was cleaned by PBS and deionized water.

### Confirmation of mitochondria

To confirm the extracted particles after isolation were indeed mitochondria, we stained cells using Rhodamine 123 (Sigma), a mitochondria specific dye reported by Johnson^1^, when the cells proliferated under the culture media in petri dishes. After isolating mitochondria as described above, we checked using a fluorescence microscope to make sure that isolated particles were indeed mitochondria (Fig. 4(e)). The mitochondrion used for the resolution enhancement experiment shown in Fig. 4 was stained by this procedure. The mitochondrion used for the 3D CXDI visualization, however, was not stained in order to obtain natural electron density of a mitochondrion exactly.

### Data acquisition for 3D tomography of a mitochondrion

Coherent x-ray diffraction measurements for 3D visualization of a mitochondrion were carried out at BL29XU of SPring-8. The coherent portion of the 5 keV (2.48 Å) incident x-ray beam was isolated by a 10 *μ*m pinhole and a pair of slits made of silicon were used to eliminate parasitic scattering from the pinhole. An isolated mitochondrion placed on a 30 nm thick Si_3_N_4_ membrane (Silson Ltd.) was illuminated by the x-ray beam and the diffraction pattern was recorded by an x-ray CCD (PI-LCX, liquid nitrogen cooling type, Princeton Instruments) in the transmission geometry. The experiment was performed in vacuum (10^−3^ Torr) to minimize air scattering background. The mitochondrion used for the 3D x-ray imaging was not dyed.

Diffraction signal was measured at 26 projection angles, 0°, ±7.13°, ±14.04°, ±20.56°, ±26.57°, ±32°, ±36.87°, ±41.19°, ±45°, ±48.81°, ±53.13°, ±58°, ±63.43°, and 69.44° to reconstruct 3D architecture of a mitochondrion using the tomography principle. To minimize the radiation damage, the angles were selected according to the EST method. To record meaningful diffracted signal with minimal acquisition time at each angle, we measured diffraction signal in low and high diffraction angle regions separately. In the low angle measurement, we blocked the main beam using polished Tantalum blades which also blocked several pixels of the zeroth order speckle. During the measurement in the high angle region, a wider area around the central speckles was blocked to increase the exposure time without having any pixel saturated. A single mitochondrion was used for the measurements at all angles. It took about 26.5 minutes on average to acquire one set of diffraction patterns at each angle. The total exposure time was 689.8 minutes.

### Diffraction measurement of a mitochondrion with reference objects

The measurements were also performed at BL29XU of SPring-8. Incident beam of 6 keV (2.07 Å) was focused by K-B mirrors to increase beam intensity to 50 times higher than the unfocused beam. Au reference objects were fabricated by coating Au film using electron beam evaporation on a pre-shaped resist on a Si_3_N_4_ membrane patterned by electron beam lithography. The final form of the reference objects were obtained by removing the remaining resist together with Au on top. Mitochondria were deposited exactly on the reference objects by a drop of medium containing isolated mitochondria. The existence of an isolated mitochondrion was confirmed by using a green fluorescence microscope after dying with Rhodamine 123 (Fig. 4(e)).

### Image reconstruction

To reconstruct a projection image at each angle, the diffraction data obtained in the low and high angle regions were patched by comparing the recorded amplitude in the overlapped area. Diffraction signal blocked by the beam stopper was replaced with the signal at opposite angles using the centrosymmetry. With the patched diffraction amplitude, we ran the GHIO algorithm^34^ with 16 different initial conditions for 9 generations to retrieve an electron density map in real space. Three best images with the smallest R-factor were averaged to obtain an 2D projection image. These procedures^41^ were applied to the data obtained at all 26 angles. For 3D image reconstruction, the center of all 2D projection images were aligned by matching the center of mass^42^ and the tomography principle was applied. We applied the EST algorithm^20, 21^ on the 26 images to reconstruct a 3D array of mitochondrion electron density.

### Electron density calculation

The electron density of the mitochondrion was estimated using the diffracted x-ray intensity in the central pixel of the CCD, $I\mathrm{(0)}={I}_{0}{r}_{e}^{2}{N}_{e}^{2}d{\rm{\Omega }}$ where *I*(0) is the number of diffracted x-ray photons in the central pixel, *I*_0_ is the incident x-ray flux, *r*_*e*_ is the electron scattering length, *N*_*e*_ is the number of total electrons interacted with x-rays, and *d*Ω is the solid angle covered by the pixel^43^. The mitochondrial electron density was estimated to be order of 10^7^ electrons/voxel although it depends on the microscopic position within the specimen. The size of a single voxel, which was determined by the maximum spatial frequency value, was (36 nm)^3^.

### Radiation dose estimation

For the 3D imaging of mitochondrion, extended exposure was necessary as there were as many as 26 projection angles. To insure that the measured sample was not damaged by the x-ray radiation, we estimated the radiation dose on the sample. The incident x-ray flux (F) was 3.6 × 10^6^ *photons*/*μm* ^2^/*s* and total exposure time was 689.9 minutes. The specimen density (*ρ*) was 1.322 g/cm^3^ and the linear absorption coefficient (*μ*) is 47.192 /cm assuming that the composition of mitochondrion is similar to that of a protein with empirical composition H_50_C_30_N_9_O_10_S_1_^25, 44^. The total dose was calculated by *D* = *FμEt*/*ρ* where *E* is incident x-ray energy and *t* is total exposure time. From this equation, the amount of radiation on the mitochondrion was calculated to be 4.26 × 10^8^ *Gy*.

### Mass density calculation

The mass density of the mitochondrion is calculated by *ρ*_*mass*_ = *ρ*_*e*_ *M*_*w*_/*Vn*_*e*_ *N*_*A*_ where *ρ*_*e*_ is the average electron density of mitochondrion, *n*_*e*_ is the number of electrons per molecule, *M*_*w*_ is molecular weight, *V* is mitochondrial volume calculated from the retrieved mitochondrion, and *N*_*A*_ is Avogadro’s number. We assumed that the mitochondrion was composed of H_50_C_30_N_9_O_10_S_1_^25, 44^, and *n*_*e*_ and *M*_*w*_ is 389 electrons/molecule and 728 g/mol respectively. The calculated mass density *ρ*_*mass*_ is 1.36 g/cm^3^. Reported values of mitochondrial mass density range from 1.09 to 1.25 g/cm^3^ depending on their phases, species and grown medium condition^45, 46^. The difference between the density obtained from the CXDI image and the value obtained during the mitochondria separation was possibly due to some unwanted particles included in the specimen and the reconstruction error.

### 3D resolution estimation

The 3D resolution was estimated by evaluating the PRTF at a given spatial frequency $\overrightarrow{q}$ using $\sum S{(\overrightarrow{q})}_{recon}{e}^{i\varphi {(\overrightarrow{q})}_{recon}}/\sum S{(\overrightarrow{q})}_{recon}$ where $S{(\overrightarrow{q})}_{recon}$ is the reconstructed diffraction amplitude and $\varphi {(\overrightarrow{q})}_{recon}$ is the retrieved phase. The sum was over 10 independent reconstructions. The PRTF value was evaluated at all measured $\overrightarrow{q}$s in all projection angles with a fixed magnitude which were averaged to plot it as a function of $|\overrightarrow{q}|$ as shown in Supplementary Fig. S3. PRTF decreases with increasing $|\overrightarrow{q}|$ and saturated at the dotted vertical line which corresponds to a resolution of 60.3 nm. The saturated PRTF value was larger than the widely used criterion of 1/*e* or 0.5.

## Electronic supplementary material

Animation of sectioned images

Supplementary Information
