# Supplementary material for: Visualization of a Mammalian Mitochondrion by Coherent X-ray Diffractive Imaging
Source: Sci Rep. 2017 May 12;7:1850. doi: 10.1038/s41598-017-01833-x (PMC5431869; doi:10.1038/s41598-017-01833-x)
Supplement: Supplementary file 2 — Supplementary Information [file 41598_2017_1833_MOESM2_ESM.pdf]

Supplementary Information

Visualization of a Mammalian Mitochondrion by Coherent X-ray Diffractive Imaging

Yoonhee Kim[1], Chan Kim[1,2], Ouyoung Kwon[1], Daewoong Nam[3], Sang Soo Kim[4], Jae Hyun Park[4], Sunam Kim[4], Marcus Gallagher-Jones[5,6], Yoshiki Kohmura[5], Tetsuya Ishikawa[5], Changyong Song[3,5], Giyoong Tae[1], and Do Young Noh[1,*]

[1]Department of Physics and Photon Science & School of Materials Science and Engineering, Gwangju Institute of Science and Technology, Gwangju 61005, Korea

[2]European XFEL, Schenefeld, 22869, Germany

[3]Department of Physics, Pohang University of Science and Technology, Pohang 37673, Korea

[4]Pohang Accelerator Laboratory, Pohang University of Science and Technology, Pohang 37673, Korea

[5]RIKEN SPring-8 Center, Kouto 1-1-1, Hyogo 679-5148, Japan

[6]Department of Physics and Astronomy and California NanoSystems Institute, University of California Los Angeles, California 90095, USA

[*]dynoh@gist.ac.kr

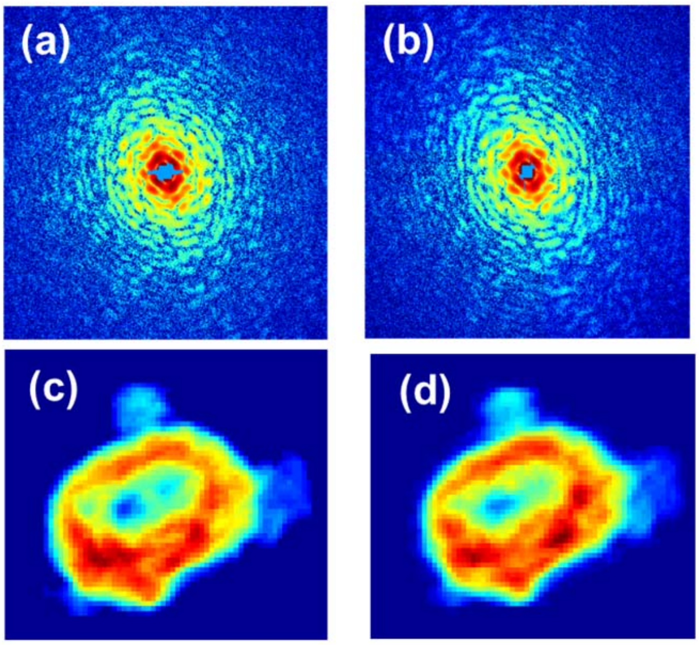

Supplementary Figure S1.

(a) Coherent x-ray diffraction pattern at zero degree projection angle (b) Diffraction pattern after 470 min of x-ray exposure (c, d) Two dimensional projection CXDI images reconstructed from (a) and (b) respectively. There was no significant difference either in the diffraction pattern or reconstruction image.

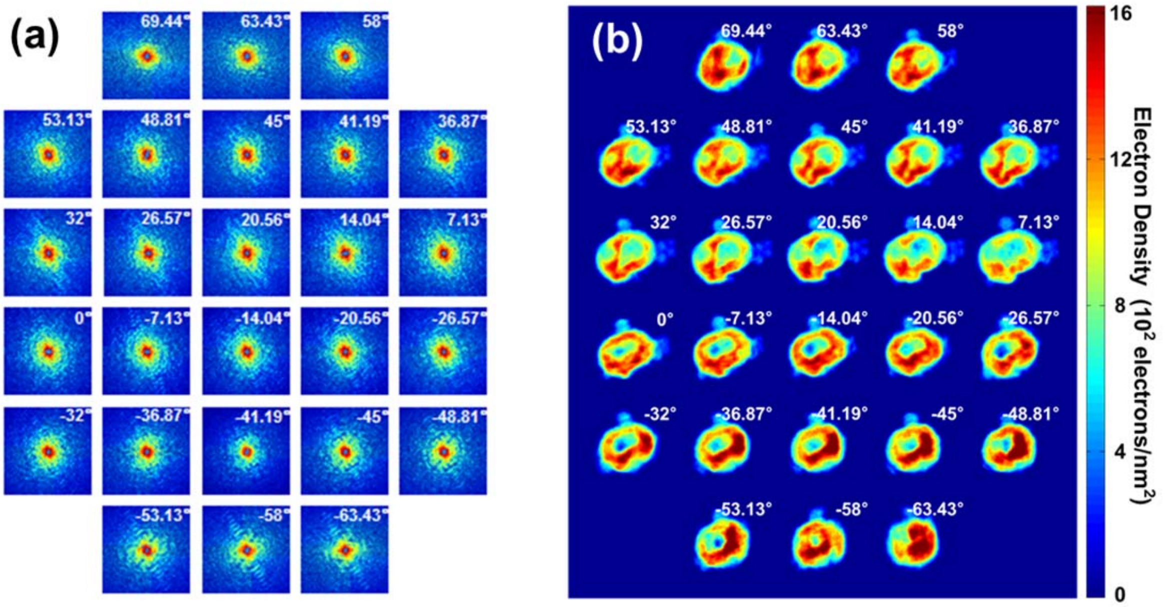

Supplementary Figure S2.

(a) Coherent x-ray diffraction pattern obtained at all 26 projection angles. (b) Two dimensional projection CXDI images reconstructed from the data shown in Figure S1. They represent the electron density map integrated along the main beam direction at corresponding projection angles.

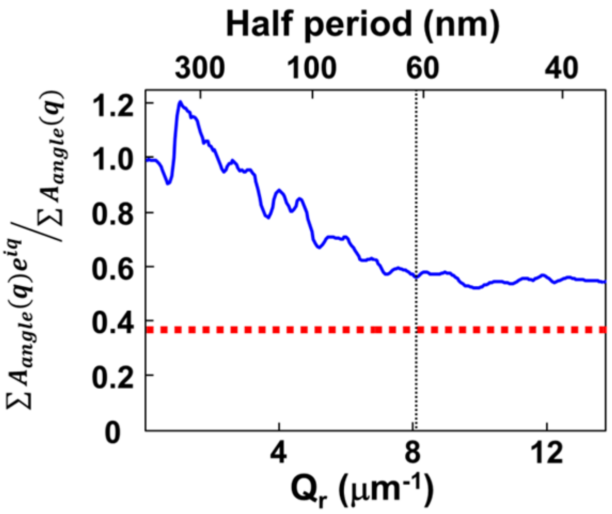

Supplementary Figure S3.

PRTF evaluated from the 3D reconstruction by $\sum S(\boldsymbol{q})_{recon} e^{i\varphi q_{recon}} / \sum S(\boldsymbol{q})_{recon}$. The value stays above 1/e level indicated by the red horizontal dotted line. The position marked by the black dotted vertical line where the PRTF saturates was quoted as the 3D resolution of 60.3 nm.

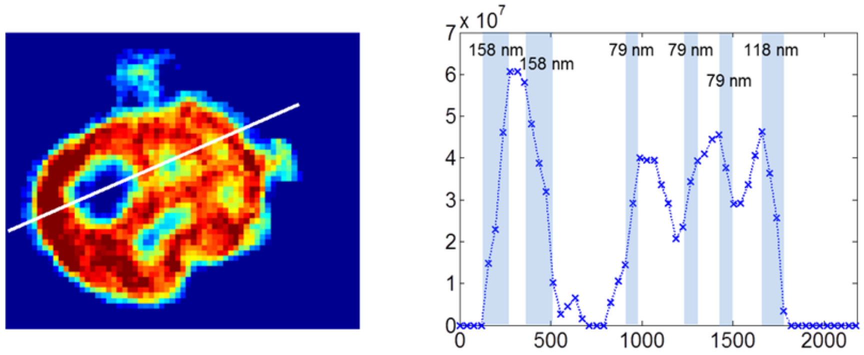

Supplementary Figure S4.

A line profile through the sectioned image shown in Fig. 2(c) to illustrate the image resolution in 3D. There are several features smaller than 100 nm.

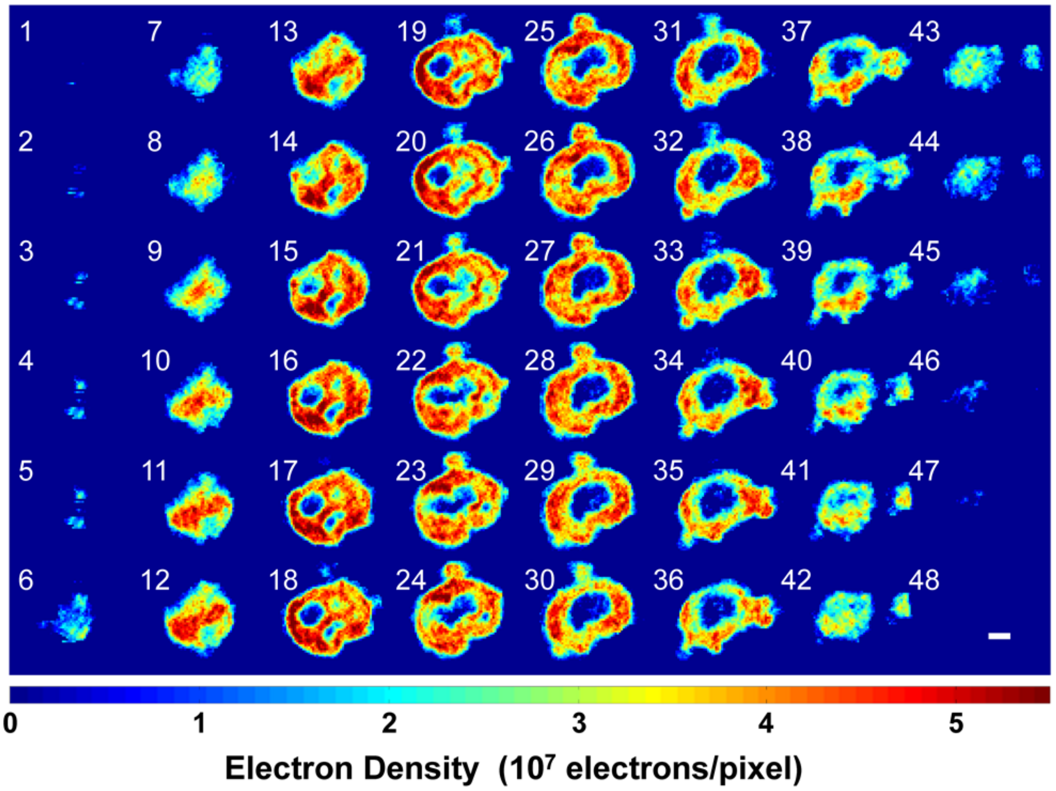

Supplementary Figure S5.

Sectioned images of the 3D mitochondrion tomogram cut in the y-z plane obtained at 36.33 nm intervals which corresponds to the single pixel size. The numbers marked on the upper left side of the each image indicate the distance from the edge in the unit of the single pixel size. Images 15-18 show baffle shape while images 19-25 look similar to septa shape. Cristae located near the perimeter of mitochondrion are observed in images 26-40.

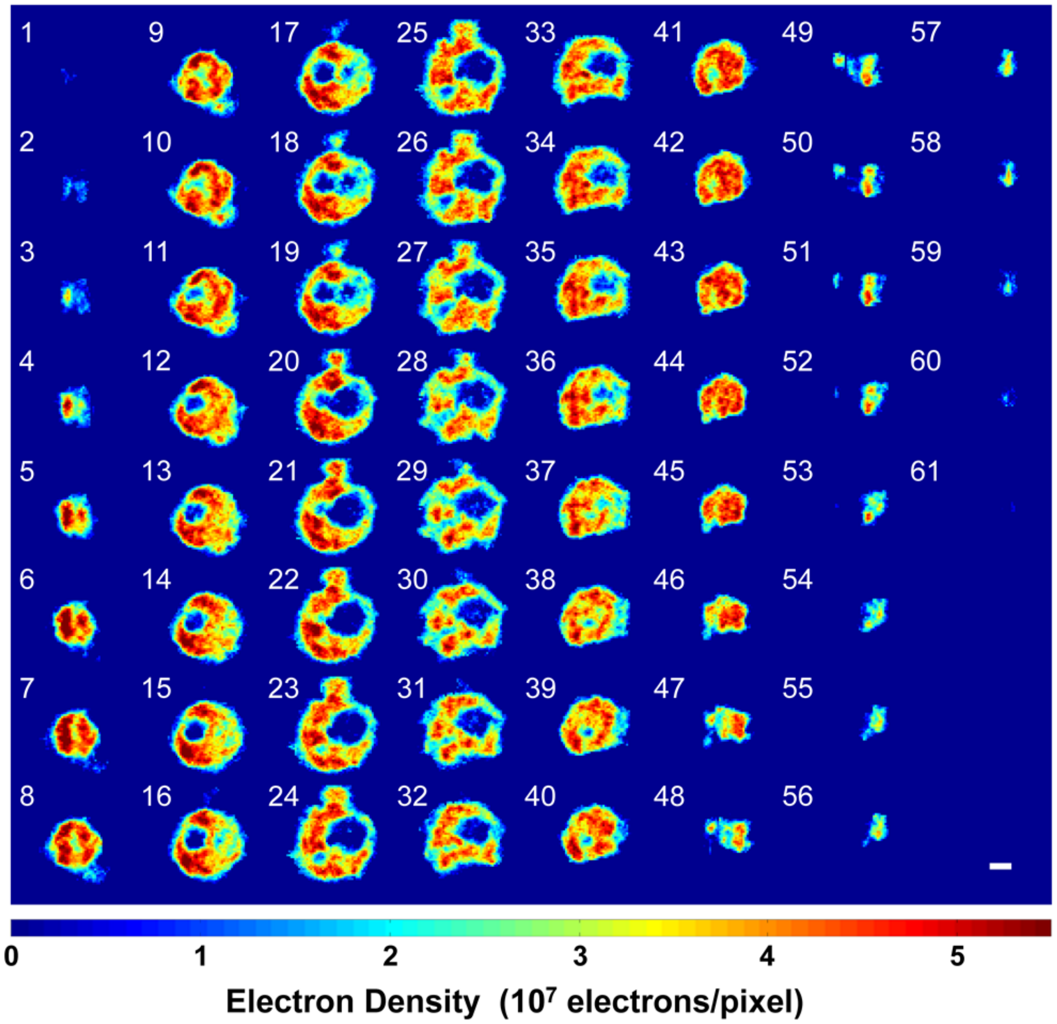

Supplementary Figure S6.

Sectioned images of the 3D mitochondrion tomogram cut in the x-z plane obtained at 36.33 nm intervals. In this direction, mitochondrion is rather circular in shape. The structural variations in the outer region are shown in most images. Low density mitochondrial matrix is localized to a circular (or spherical) region.

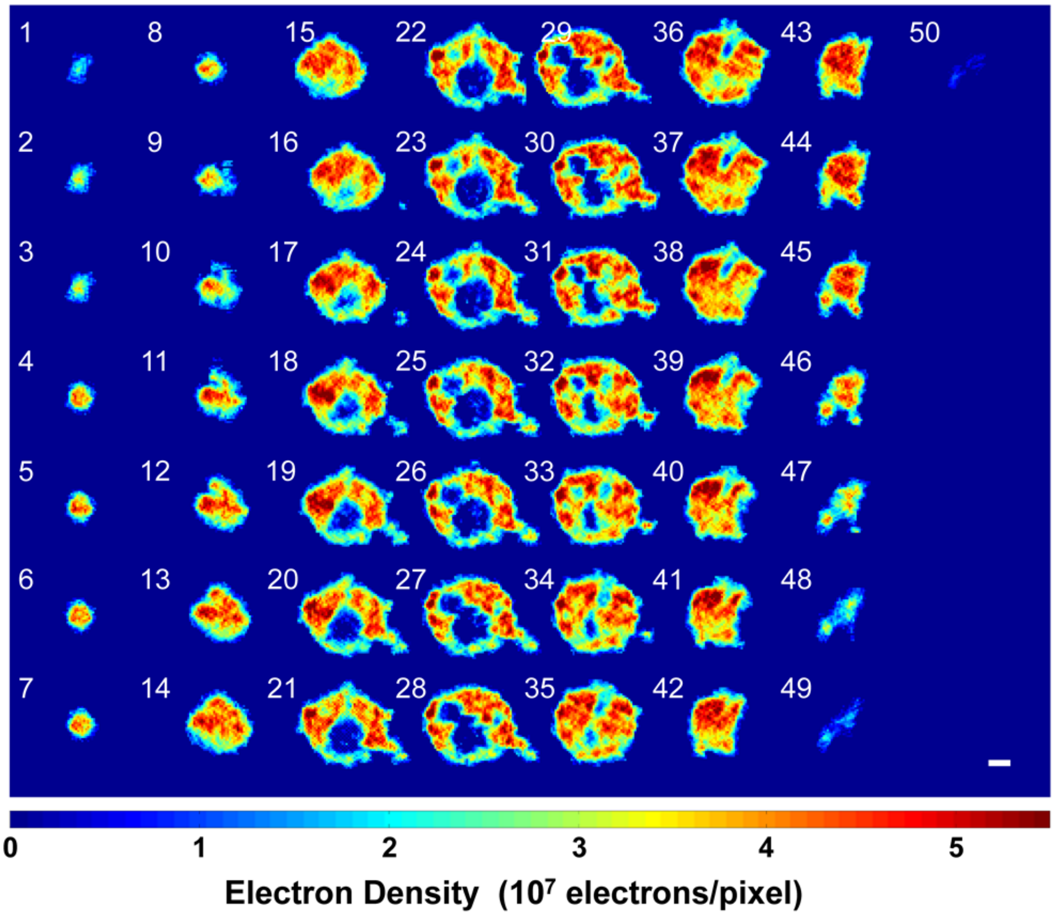

Supplementary Figure S7.

Sectioned images of the 3D mitochondrion tomogram cut in the x-y plane obtained at 36.33 nm intervals. Complicated internal structural variations are illustrated in the images.

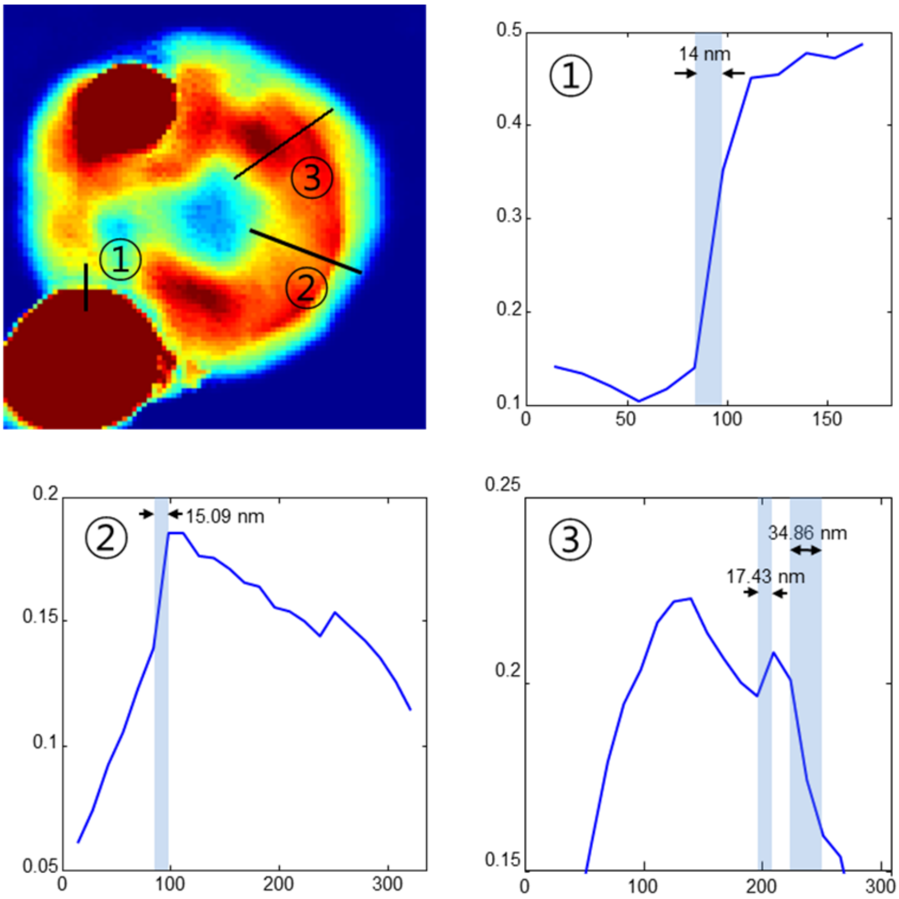

Supplementary Figure S8.

A line profile through the mitochondria projection image with reference Au objects shown in Fig. 4(b). The image resolution 14 nm is reflected in the line through the edge a Au particle. However, the detailed mitochondrial structures are not clearly resolved since the image is a projected image where all densities are summed along the beam direction.

Supplementary Movie S9. Animations illustrating the sequential sectioned images cut in the y-z, x-z, and x-y direction. Three dimensional morphology of the mitochondrion is well described. The overall mitochondrial shape is illustrated using translucent green.
